# Enhanced CATBraTS for Brain Tumour Semantic Segmentation

**DOI:** 10.3390/jimaging11010008

**Published:** 2025-01-03

**Authors:** Rim El Badaoui, Ester Bonmati Coll, Alexandra Psarrou, Hykoush A. Asaturyan, Barbara Villarini

**Affiliations:** School of Computer Science and Engineering, University of Westminster, London W1W 6UW, UK; e.bonmaticoll@westminster.ac.uk (E.B.C.); a.psarrou1@westminster.ac.uk (A.P.); h.asaturyan1@westminster.ac.uk (H.A.A.)

**Keywords:** brain tumour, convolutional neural network, semantic segmentation, transformer, tumour segmentation

## Abstract

The early and precise identification of a brain tumour is imperative for enhancing a patient’s life expectancy; this can be facilitated by quick and efficient tumour segmentation in medical imaging. Automatic brain tumour segmentation tools in computer vision have integrated powerful deep learning architectures to enable accurate tumour boundary delineation. Our study aims to demonstrate improved segmentation accuracy and higher statistical stability, using datasets obtained from diverse imaging acquisition parameters. This paper introduces a novel, fully automated model called Enhanced Channel Attention Transformer (E-CATBraTS) for Brain Tumour Semantic Segmentation; this model builds upon 3D CATBraTS, a vision transformer employed in magnetic resonance imaging (MRI) brain tumour segmentation tasks. E-CATBraTS integrates convolutional neural networks and Swin Transformer, incorporating channel shuffling and attention mechanisms to effectively segment brain tumours in multi-modal MRI. The model was evaluated on four datasets containing 3137 brain MRI scans. Through the adoption of E-CATBraTS, the accuracy of the results improved significantly on two datasets, outperforming the current state-of-the-art models by a mean DSC of 2.6% while maintaining a high accuracy that is comparable to the top-performing models on the other datasets. The results demonstrate that E-CATBraTS achieves both high segmentation accuracy and elevated generalisation abilities, ensuring the model is robust to dataset variation.

## 1. Introduction

A brain lesion is an abnormality in the brain caused by injury or disease, and such lesions can disrupt communication in the affected area. Brain lesions take many forms and vary in severity depending on their types and causes [1]. For example, some lesions are caused by traumatic brain injury, which can lead to strokes, infections in the brain, decreased cognitive function, and brain tumours [2].

Clinically defined as a cerebral neoplasm, a brain tumour is formed by abnormal and excessive growth of mutated tissues within or near a brain [3]. Moreover, primary brain tumours originate in the brain, whereas secondary brain tumours can spread to the brain from other organs such as the lungs, colon, and kidneys. A benign brain tumour is non-cancerous and grows relatively slowly in the brain. Symptoms might progressively worsen over a matter of months or years, which include drowsiness, nausea, and vomiting, and persistent headaches. In contrast, malignant brain tumours are fast-growing and cancerous, and a sufferer may quickly develop symptoms over days or weeks [4]. Specialists develop treatment plans based on factors such as the location and aggressiveness of the tumour, which is indicated by its grade and the patient’s age and sex [5].

The mortality rate for brain tumours is the second highest of all major cancers, with a five-year survival rate of 12%. The highest is pancreatic cancer. There are several factors behind the low survival rate [6]; they include the misdiagnosis or late detection of malignant brain tumours. A recent analysis of extensive patient data concluded that a four-week delay in appropriate treatment is associated with increased mortality [7].

Manual brain tumour detection in medical images such as magnetic resonance imaging (MRI) volumes is a tedious, time-consuming task and subject to the limitations of human eyesight. An investigation into radiologists’ workloads [8] revealed that they would have to read around one medical image scan every 3 s in a work shift to deal with the overload. Hence, a significant amount of the literature on deep learning (DL) networks focuses on medical image analysis, particularly in developing precise computer-aided diagnosis (CAD) tools for tumour segmentation.

As an architecture for DL algorithms, convolutional neural networks (CNNs) have dominated CAD-based medical image analysis by outperforming artificial neural networks (ANN) and long short-term memory (LSTM) for automatic 3-dimensional (3D) organ segmentation [9] and classification in medical images; they have resulted in higher accuracy relative to the gold standard of expert-led manual segmentation [10,11]. For instance, CNNs can be employed to classify interstitial lung disease patterns of the lung [12], skin cancer detection [13], and fundus detection [14]. However, one major drawback of this architecture is the application of attention mechanisms, which only focus on neighbouring pixels and do not relate to global features. Thus, transformers were introduced as a self-attention approach for overcoming this issue, in which every element can link to long-range dependencies; this approach received exceptional interest after surpassing competing CNNs in natural language processing (NLP) tasks [15]. Following the tremendous success of the transformers for NLP, researchers from Google considered applying transformers for computer visioning and proposed an architecture named Vision Transformer (ViT) [16]. In this paper, we propose an Enhanced Channel Attention Transformer for Brain Tumour Semantic Segmentation (E-CATBraTS) as a novel state-of-the-art DL model for brain tumour segmentation in multi-modal MRI volumes. We trained and evaluated E-CATBraTS on four datasets, demonstrating that our novel approach outperforms the current state-of-the-art models in accuracy and generalisability. The implementation is available at https://github.com/RimElBadaoui/E-CATBraTS, (accessed on 31 July 2024). The original contributions to research in the context of medical image segmentation are as follows:

- A novel ViT-CNN model for the automatic segmentation of brain tumours in MRI volumes, which uses channel shuffling and a channel-attention mechanism; this improves both segmentation accuracy and the model’s generalisability on different MRI sequences and multi-modal MRI datasets;
- An original CNN encoding block with a channel-attention module, which can exploit tumour features and optimise the robustness of the segmentation on various brain tumour regions and image artefacts;
- A comprehensive validation on four different datasets, which demonstrates higher segmentation accuracy and generalisability, compared to the current state-of-the-art models.

The remainder of this paper is organised as follows. Section 2 provides a brief overview of current state-of-the-art DL models for medical image segmentation. Section 3 details the proposed E-CATBraTS model for brain tumour segmentation, while Section 4 describes the four different datasets used for training, validating and evaluating the proposed approach; it covers the evaluation and the implementation details. Section 5 delivers and performs a quantitative analysis of the segmentation results. Moreover, Section 6 discusses and provides a critique of these results against the current state-of-the-art models. In conclusion, Section 7 provides a summary of the proposed novel approach, coupled with findings and future work.

## 2. Background

Over the past decade, different types of CNN architectures have dominated the field of radiomics in modern medicine [17], including the successful integration of an attention mechanism layer. Most recently still, ViTs have gained popularity as a method for solving medical image classification and segmentation tasks.

One of the most widely used CNNs is known as U-Net, which is based on the fully convolutional network [18,19]. The architecture primarily consists of a downsampling (encoder) and upsampling (decoder) phase. The main contribution of U-Net is presented in the decoder network consisting of four blocks, in which convolutional upsampling replaces the max pooling layers to enhance the resolution of the output. U-Net suffers from the vanishing gradient problem: increasing the number of layers causes the gradients of the loss function to decrease exponentially as it propagates down to the initial input layer and leads to degradation in network convergence.

Another encoder–decoder architecture, SegResNet, builds upon ResNet with an auto-encoder (VAE) branch to address the problem of vanishing gradient [20,21]. First, the encoder network extracts feature maps from the image using residual blocks. Secondly, the decoder employs a 3D 1×1 convolutional layer and 3D bi-linear upsampling to increase the maps’ spatial size, which is then added to the output of the equivalent encoding block. The output classes are computed by a 3D 1×1 convolutional layer and a sigmoid activation function. Despite winning the RSNA-ASNR-MICCAI Brain Tumor Segmentation (BraTS) Challenge of 2018 [22], the SegResNet architecture adds more complexity to the training phase, leading to an over-fitted model [23].

In the past year, the novel architecture Shifted WINdows UNEt TRansformers (Swin UNetR) has gained popularity as a ViT neural network for 3D semantic segmentation of brain tumours in MRI volumes. Swin UNetR is based on the Swin Transformer (Swin-T) merged with a CNN-based decoder utilising a shifted window module [24].

Swin UNetR passes the network’s input to Swin-T, which creates 3D non-overlapping tokens of the input using a patch-splitting mechanism. By employing a transformer encoder, Swin-T reduces the number of patches in one of the four stages; the first stage of the transformer encoder consists of a linear embedding layer and two transformer blocks based on shifted windows [25]. Each subsequent stage contains a patch merging layer to downsize the features by a factor of 2 and two transformer blocks.

Next, the encoded features pass through a CNN residual decoder that upsamples the features to the original resolution via skip connections. Swin UNetR performed better than current state-of-the-art models, such as SegResNet, nnU-Net [26,27] and TransBTS [28], in the BraTS 2021 challenge validation phase. However, the findings could have been more comprehensive if Swin UNetR had been tested on datasets with varying image quality. The performance of Swin UNetR is likely to decline when applied to lower-quality images, which restricts its use to high-quality datasets. It is essential to develop a model that remains robustly accurate across different imaging qualities so that it can be used effectively in real-life scenarios where medical images come in various resolutions.

## 3. Methodology

**Figure 1:**
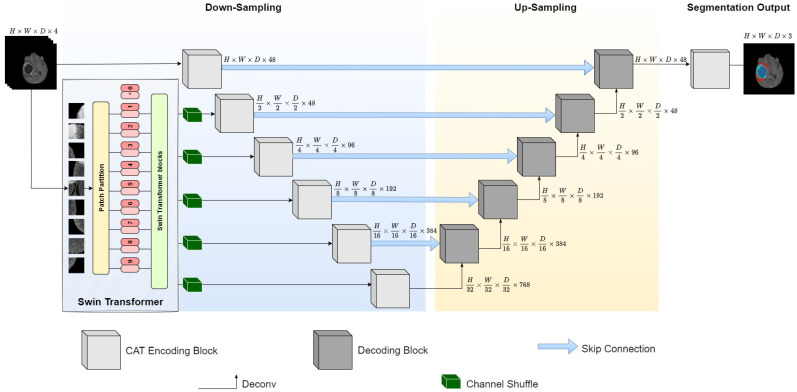
E-CATBraTS with channel shuffle module for shuffling embedded feature maps prior to reducing its size using a novel CAT encoding block. The blue background represents the encoder, while the yellow represents the decoder.

In a real-life scenario, the quality of medical imaging varies for several reasons. For example, the slightest movement by a patient during their scanning process will add artefacts known as noise to the resultant image and negatively impact its interpretation. The most common noises include Gaussian, salt and pepper, poison and impulse [29]. Therefore, producing a generalisable model that can be successfully applied to multiple datasets with high accuracy is essential. For this purpose, we developed E-CATBraTS, a novel model that improves upon 3D CATBraTS [30] and delivers higher generalisability and segmentation accuracy across datasets acquired using multiple sequences and scanner protocols. 3D CATBraTS is a hybrid deep learning method that employs ViTs and CNNs for 3D brain tumour segmentation in MRI and has outperformed competing methods in the validation phase of the BraTS 2021 challenge. However, the main weakness of the approach is its reliance on specific data, which makes it susceptible to biases and results in poorer segmentation performance when dealing with new, unseen data. To address these issues, our proposed model, E-CATBraTS, employs channel-shuffling and channel-attention mechanisms and can be described in three main parts, namely the Swin Transformer, the down-sampling, and the up-sampling, as shown in Figure 1.

**Figure 2:**
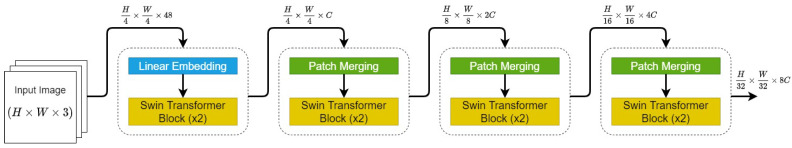
Swin Transformer with four stages: it takes, as an input, non-overlapping patches of magnetic resonance imaging (MRI) volumes.

The first part integrates the Swin Transformer (Swin-T), as illustrated in Figure 2, which processes a multi-modal MR image input with dimensions 128×128×96×4 and splits the volume into non-overlapping shifting windows using a patch partition module. Shifted windows allow improved efficiency by computing self-attention from local windows, and its hierarchical structure ensures scalability to compute information at different scales. After splitting, the patches undergo linear embedding to produce patches of size 2×2. Next, two transformer blocks are applied to the tokens to complete the first stage. The resulting output is processed through the second stage, and each additional stage contains a patch merging layer that concatenates neighbouring patches and downsamples the number of patches by a factor of 2, followed by two transformer blocks employed for feature transformation.

**Figure 3:**
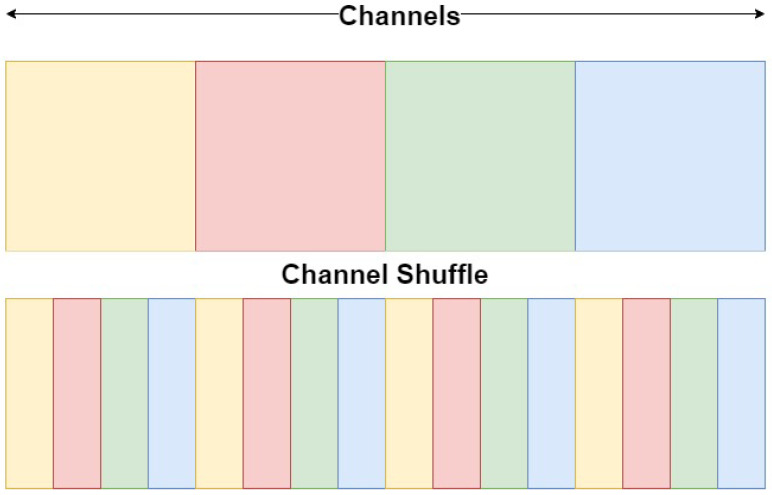
Channel shuffle. Channels are divided into four subgroups. Yellow, red, green, and blue represent the channels of the four MRI acquisitions: T1, T1-weighted, T2, and T2-FLAIR.

In the second part of E-CATBraTS, we introduce five channel shuffling blocks applied to the output of Swin-T, shown as green blocks in Figure 1. Consider an input of size W×H×C to represent the width, height and number of channels, respectively. Channel shuffling is a computationally effective operation that reshapes the feature map as $W\times H\times G\times \frac{C}{G}$, where G denotes the number of groups in which to divide the channels. Next, the tensor is permuted and reshaped to the original dimensions. Figure 3 illustrates the process of channel shuffling: the squares coloured yellow, red, green, and blue designate the channels of the four MRI acquisitions T1, T1-weighted, T2, and T2-FLAIR. We set the number of groups to 4 based on the number of MRI acquisitions. We also experimented with the module having two groups, allowing each channel to exchange information with one from another group in order to prevent feature loss. By shuffling the channels, we enable the flow of information between the feature maps in the same spatial location so each group holds information from the other groups. We use the channel shuffling mechanism as a regularisation technique to improve our model’s evaluation accuracy and convergence rate and help reduce the risk of overfitting. Moreover, when shuffling the feature maps between the channels, it will work as structured noise for the channels, which can substantially improve the model’s generalisable capabilities. After shuffling the feature maps, we perform downsampling using CNN encoding blocks to reduce the size of the feature maps.

**Figure 4:**
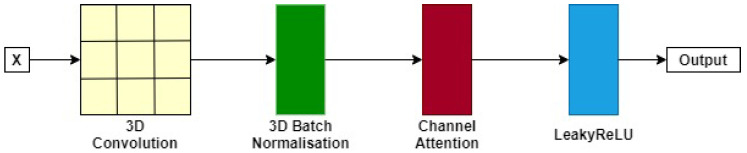
A single CAT encoding block. The block takes X as an input and applies a 3D convolution. Next, normalisation is performed using a 3D batch normalisation function before progressing through a channel attention module and activated in a LeakyReLU layer.

We removed the residual encoding blocks of 3D CATBraTS and replaced them with six novel, simplified CNN-based alternatives known as CAT blocks, as presented in Figure 4. Our proposed encoding block processes a given input, which corresponds to the output channels generated from the channel shuffling blocks, through a 3×3×3 convolutional layer of stride 1 and padding 1. The next step includes 3D batch normalisation and integration of a channel attention module of two layers: a global average pooling layer and a fully connected layer. A global average pooling layer reduces the spatial dimension of feature maps by averaging the feature maps to attain channel weights. This is computed as follows:

$$m_{k}=\frac{1}{W\times H}\sum_{i=1}^{W}\sum_{j=1}^{H}x_{k}\left(i,j\right),$$

where $x_{k}\left(i,j\right)$ represents the feature map of the *k*th channel on the spatial location (i,j) [31]. $m_{k}$ denotes *k*th channel global average pooling.

The fully connected layer obtains the cross-relationship between the channels and scales the weights. Not all feature maps exhibit the same level of importance for network optimisation; for instance, feature maps containing background information contribute less to the resultant segmentation than feature maps containing more meaningful contextual information, such as the target tumour and surrounding membrane tissue. Thus, we employed the attention module to provide extra weight to the channels that significantly exploit tumour features of interest, which will improve the convergence and generalisation capabilities of the model [32]. The output of a CAT block is a LeakyReLU of the channel attention module. It is important to note that the feature maps’ size in the first CAT block is H×W×D×48, and this is reduced by a factor of 2 until reaching $\frac{H}{32}\times \frac{W}{32}\times \frac{D}{32}\times 768$ in the final block.

In the third part of our proposed model, the feature maps are upsampled to their original size using five residual decoding blocks, as highlighted in Figure 1. Upon completion, a 1×1×1 convolution is applied to map out three tumour subclasses of interest: whole tumour (WT), tumour core (TC), and enhancing tumour (ET).

## 4. Experiments

### 4.1. Datasets

To evaluate the performance and robustness of E-CATBraTS, we trained and tested our model on four datasets: UCSF-PDGM, UPENN-GBM, EGD, and BraTS 2021

The UCSF-PDGM dataset includes 501 cases of MRI volumes, which can be accessed from the Cancer Imaging Archive [33,34,35]. The imaging modalities include T1, T1-weighted, T2, and T2-FLAIR primarily. The ground-truth labels were initially generated using a winning segmentation algorithm and manually reviewed and edited by trained radiologists; the dataset includes the labels for the enhancing tumour (ET), tumour core (TC), and whole tumour (WT). We split the dataset into 351 for training, 101 for validation and 49 cases for evaluating our proposed model.

UPENN-GBM is a collection of 611 cases of MRI volumes involving de novo Glioblastoma (GBM) patients from the University of Pennsylvania Health System (UPENN) between 2006 and 2018 [35,36,37]. The MR images were obtained via sequences T1, T1-weighted, T2, and T2-FLAIR, coupled with the techniques of diffusion tensor imaging and dynamic susceptibility contrast for most cases. In this study, we split the dataset into 427 cases for training, 122 cases for validation and 62 cases to evaluate our model’s performance.

The Erasmus Glioma Database (EGD) contains the MRI scans of patients with glioma, with 281 female cases, 492 male cases, and one unknown case [38]. The MR images were obtained using four main acquisition protocols, including T1, T1Gd, T2, and T2-Flair. A WT ground truth segmentation was included for each case, 374 of which were manually annotated before registration to a common atlas, and the remaining 400 were automatically segmented after registration. For this study, we split the EGD database into 540 cases for training, 117 cases for validation and 117 for testing our model. EGD is available at the Health-RI XNAT upon request and granted access after signing a data usage agreement [39].

The Brain Tumor Segmentation BraTS dataset for 2021 is arguably one of the most popular datasets employed in developing and testing novel brain tumour segmentation models. BraTS contains the brain MRI scans of 1251 patients diagnosed with brain tumours, all of whom underwent pre-processing and were manually annotated and reviewed by specialist radiologists. The dataset also provides the four MRI modalities: T1, T1Gd, T2, and T2-Flair [40,41,42]. We used a subset of 50 scans from the UCSF-PDGM dataset for testing.

### 4.2. Evaluation

To evaluate the segmentation accuracy of E-CATBraTS, we use the Dice similarity coefficient (DSC), Jaccard index, and Hausdorff distance (HD) performance metrics. DSC is a score between 0 and 1, which measures the similarity between the ground truth and prediction as two separate datasets. DSC can be defined as follows:

$$DSC\left(G,P\right)=\frac{2|G\cap P|}{\left|G|+|P\right|},$$

where DSC(G,P) is the overlap between G and P, representing the ground truth and the prediction, respectively.

The Jaccard index, also referred to as Intersection over Union (IoU), computes the overlap between the ground truth and prediction divided by the union of the two.

$$IoU\left(G,P\right)=\frac{\left|G\cap P\right|}{\left|G\cup P\right|},$$

The HD measures the Euclidian distance between the points of the ground truth and the segmentation set. The smaller the Hausdorff distance, the better the match between the two sets.

$$HD\left(G,P\right)=\max_{g\epsilon G}\left\{\min_{p\epsilon P}\left\{d\left(g,p\right)\right\}\right\},$$

where *g* and *p* are points in sets G and P, respectively and d(g,p) is the distance between points *g* and *p*.

### 4.3. Implementation Details

E-CATBraTS was implemented using MONAI framework [43]. All models were trained with an initial learning rate of $5\times 10^{-5}$ on NVIDIA GeForce RTX 3080, Acer, London, Uk. Next, using the CosineAnnealingLR scheduler [44], the initial learning rate is simultaneously decreased by the following equation:

$$lr_{t}=lr_{min}+\frac{1}{2}\left(lr_{max}-lr_{min}\right)\left(1+cos\left(\frac{E_{curr}}{E_{max}}\pi \right)\right),$$

where $lr_{t}$ is the learning rate computed at each validation using cosine annealing, $lr_{max}$ is the initial learning rate and $lr_{min}$ represents the minimum learning rate, $lr_{max}$ is the maximum number of epochs, $E_{curr}$ is the number of epochs since the last restart, and π = 3.14.

The CosineAnnealingLR scheduler is an effective technique for gradually reducing the learning rate during training. This scheduler facilitates a smooth adjustment, which enhances the model’s performance and convergence.

Our proposed model employs the stochastic optimisation method AdamW, which decays weight per the decoupling weight decay technique from the gradient update [45]. AdamW demonstrates improved generalisation and yields better training loss, outperforming similar methods.

We employ the Dice loss to train E-CATBraTS as it is widely used for handling imbalanced data, and it is formulated by subtracting the DSC from 1 [46].

## 5. Results

We trained and evaluated our proposed model and comparable state-of-the-art models on four MRI datasets. First, Table 1 highlights the results using the UCSF-PDGM dataset, for which we assessed the channel shuffle mechanism by dividing the channels into two and four groups known as GRP. Our results demonstrate that, in both experiments, E-CATBraTS outperformed the state-of-the-art models, achieving a mean DSC of 0.795 and a standard deviation of 0.034 (GRP = 4), which is 3.8% higher than the nearest competitor, UNETR. For each tumour subregion, E-CATBraTS raised the mean DSC to 0.722 for TC, 0.884 for WT and 0.778 for ET, a significant improvement that follows the same trend using the IoU and HD performance metrics. In contrast, applying SegResNet to the same dataset scored the lowest accuracy with a mean DSC of 0.673, including 0.651, 0.779, and 0.588 for TC, WT, and ET, respectively. Next to the model that yields the most accurate results is E-CATBraTS, achieving a mean DSC of 0.761 (GRP = 2) and highlights that E-CATBraTS significantly boosts 3D brain tumour segmentation by roughly 6% more than 3D CATBraTS, and achieves higher statistical stability with a lower standard deviation to indicate enhanced robustness.

**Figure 5:**
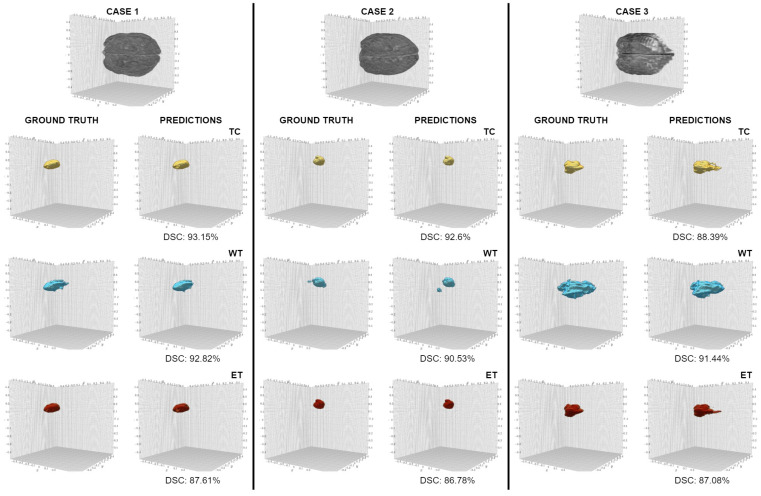
Brain tumour subregion segmentation in three randomly selected MRI cases from the test UCSF-PDGM dataset. The tumour subcategories: tumour core (TC), whole tumour (WT), and enhancing tumour are highlighted in yellow, blue, and red, respectively.

The performance of models UNETR and Swin UNeTR on the UCSF-PDGM dataset were 0.757 and 0.749 in mean DSC, respectively. Figure 5 highlights the results of E-CATBraTS in three cases with brain tumours of varying size, shape, and location. For each case, the top row depicts an image of the 3D brain reconstruction in the MRI scan, and for every tumour subclass, the subsequent left and right columns compare the ground truth and the predictions of our proposed model, respectively.

**Figure 6:**
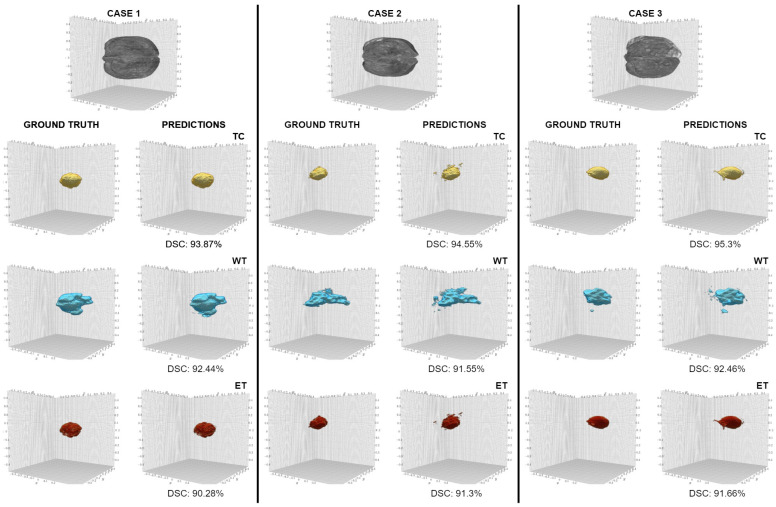
Segmented brain tumours in three randomly selected cases in the test UPENN-GBM dataset. Tumour core (TC) is marked in yellow, whole tumour (WT) in blue, and enhancing tumour (ET) in red.

Using the UPENN-GBM dataset, our proposed model yielded the best results in mean DSC, as presented in Table 2. Given two subgroups in the channel shuffle, E-CATBraTS achieved the highest segmentation accuracy for TC (0.857) and ET (0.856) and surpassed the performance of 3D CATBraTS by 4.7% overall. In contrast, the CNN-based SegResNet trailed behind with the lowest score, a mean DSC of 0.751. They were followed by our proposed method with four subgroups, UNETR and the Swin UNetR, with mean DSC: 0.850, 0.856, and 0.857, respectively. Figure 6 highlights different evaluations of E-CATBraTS in three cases.

Using the EGD dataset, E-CATBraTS performed comparably to Swin UNetR as shown in Table 3, in which case the latter scored less than 0.5% more than the former on WT, achieving a mean DSC of 0.784. Our results show that E-CATBraTS scored an overall higher accuracy than 3D CATBraTS in mean DSC of 0.780 and 0.732, respectively.

As highlighted in Table 4, our results indicate that 3D CATBraTS yielded better results on the BraTS 2021 dataset than E-CATBraTS and other state-of-the-art models. When comparing 3D CATBraTS to our proposed methodology, the former performed roughly 3% better in mean DSC accuracy (0.809) than the latter (0.770).

With respect to the other models, E-CATBraTS has outperformed both SegResNet and Swin UNeTR by around 2% when compared to the nearest model.

Evaluating the performance of E-CATBraTS with HD metric shows a lower score than the other models on UCSF-PDGM, UPENN-GBM, and EGD datasets, indicating that our proposed model has better segmentation of the tumour boundaries and its components.

Moreover, several ablation studies were conducted to demonstrate the importance of the Channel shuffling and channel attention modules in improving the generalisation capabilities and segmentation accuracy of E-CATBraTS. The results were compared to E-CATBraTS on the four datasets and evaluated using DSC, IoU, and HD metrics. The results of the ablation experiments are shown in Table 5. It is clear from the results that the addition of the channel shuffling and channel attention modules has increased the accuracy and stability of the model, which was validated using the three evaluation metrics.

## 6. Discussion

Our main goal in developing E-CATBraTS is to precisely identify and segment 3D brain tumours in MR images that could be affected by artefacts. We also aim to ensure that our proposed DL model exhibits statistical stability when applied to various datasets acquired through different imaging protocols. As such, we evaluated E-CATBraTS on four datasets generated at multiple medical centres: UCSF-PDGM, UPENN-GBM, EGD, and BraTS 2021. To the best of our knowledge, this is the first study to develop and apply a DL model on the aforementioned multi-site, multi-modal brain MRI datasets.

The results of this study, as presented in Section 5, demonstrate that E-CATBraTS outperforms the current state-of-the-art methods, including Swin UNetR and the leveraged 3D CATBraTS approach. Our proposed novel model generates more robust segmentations across all tested datasets compared to the other competing models. There are several explanations for this outcome: first, we perform channel shuffling for the embedded patches prior to downsampling. Channel shuffling was initially introduced to help reduce computational costs in object detection tasks involving mobile applications with insufficient resources [47]. Thus, when integrated into our proposed model, the shuffling operates as a DL network regulariser, enabling the cross-flow of contextual information between the T1, T1Gd, T2, and T2-Flair channels by randomly swapping their patches, which improves the model’s generalisation capabilities. Each MRI modality provides excellent soft-tissue contrast to highlight tumour subregions of interest, primarily as the intersection between the modalities generates more precise insight into the tumour subregions.

Furthermore, the proposed enhanced CAT encoding block supports our novel model, achieving higher segmentation accuracy and statistical stability. This block contains a single 3D convolution that is normalised using the batch function and then processed through a channel attention block. Employing a channel attention module has significantly improved the accuracy and robustness of E-CATBraTS without adding expensive computations to the model. As mentioned in Section 3, the channels have a different impact on each class. Accordingly, we weigh each channel based on its contribution using an average pooling layer followed by two fully connected layers.

Overall, the accuracy of our proposed model’s predictions is impacted by the characteristics of the brain tumour and the quality of the input MR image. Brain tumours vary significantly in shape, size, and location, influenced by several factors such as the tumour type and grade and patient age and sex. Such variation and inconsistency in structure and position further challenge the DL model’s ability to identify the tumour of interest accurately. Moreover, the extent to which an MR image exhibits artefacts plays a vital role in the reliability of the resultant segmentation prediction. The quality of an input MR image relies upon, but is not limited to, image acquisition, storage and transmission processes. Artefacts can also hinder the reliability of an MRI case, including image anomalies caused by software, hardware, pulse sequences or patient movement.

**Figure 7:**
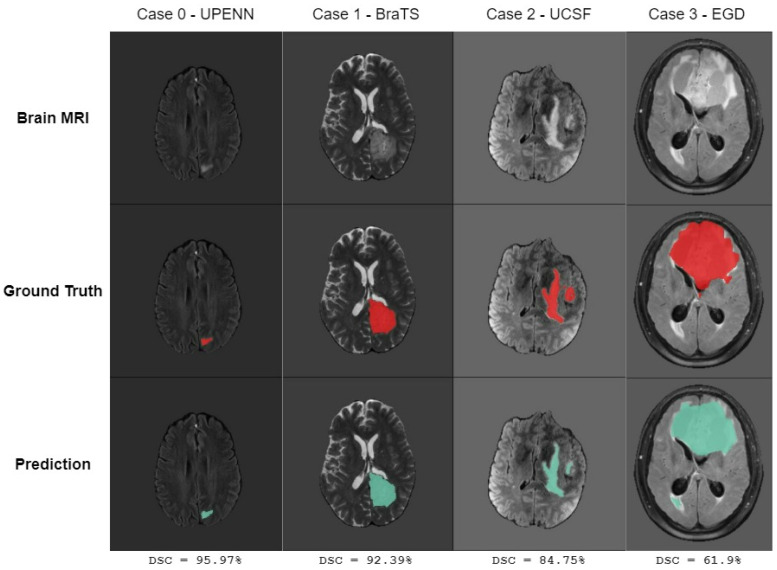
Four cases randomly taken from various datasets with different image quality. For each case, we show at the top row the original brain MRI slice; in row 2, we have the ground truth contoured in red, and in the last row, we show the prediction of the E-CATBraTS model coloured in green with the Dice similarity coefficient (DSC).

Figure 7 highlights E-CATBraTS’ resultant segmentation in a randomly selected case from each of the four datasets, illustrating the variation in image quality across the datasets. While the top row shows a single slice from an MRI brain scan, the middle and bottom row shows the ground truth and our proposed model’s prediction, respectively. Typically, bigger tumours, as in Case 3, see Figure 7, achieve higher segmentation results than their smaller counterparts, as in Case 0. Contrary to expectations, the latter outperforms in accuracy compared to the former, scoring 95.97% DSC versus 61.9% DSC. Such an outcome underlines the image quality of the datasets in question, with UPENN containing scans with lower image degradation than the EGD dataset.

The results obtained from the ablation study clearly demonstrate the contribution of the proposed techniques: the channel shuffle and the channel attention mechanisms. As shown in Table 5, the model’s accuracy significantly improves when both components are included. Conversely, the model performs poorly across all datasets when either or both components are omitted. This can be attributed to the positive impact of channel shuffling, which enhances the model’s convergence and enables it to learn more detailed features. Additionally, the channel attention mechanism allows the model to focus on the most relevant features, as previously mentioned.

As detailed in this paper, our findings carry significant implications for developing DL models tailored towards other medical imaging tasks, such as segmenting breast lesions, bone tumours and organ tumours such as lung and pancreatic cancer. The generalisability of the E-CATBraTS model could also extend towards heart segmentation to assess the risk of cardiovascular diseases and detect various fetal cardiac anomalies.

Having trained and evaluated E-CATBraTS across four different datasets under the same experimental setup, we have shown that our novel model achieves robust and statistically stable results, potentially forming the basis for an investigation into other biomedical image segmentation tasks and employed by the clinical research community to assess and better understand graded brain tumours using large-scale MRI data.

Although the study has successfully achieved higher accuracy and shown improved generalisability across various datasets, it is important to acknowledge certain limitations. To better reflect real clinical scenarios, other brain imaging protocols such as CT scans, which are a more cost-effective alternative to MRIs, should have been considered. However, this was not feasible due to the limited availability of public resources that provide labelled CT scan datasets. Additionally, there is a need for more diverse datasets to prevent biases against specific tumour types.

## 7. Conclusions

This paper proposes E-CATBraTS, a novel deep learning model for a 3D brain tumour segmentation model in MRI volumes. Our model improves upon 3D CATBraTS, which originally outperformed state-of-the-art methods in the BraTS 2021 challenge validation phase. E-CATBraTS was trained on four different datasets to exploit contextual information by using channel shuffling, thereby enhancing the information exchange and interaction between feature channels. This component allows the network to learn more complex information while also helping to prevent overfitting. In addition to channel shuffling, we incorporated a channel attention module in the encoding block, which assigns weights to each channel based on its importance. This mechanism enables the network to focus on the most significant features, ultimately optimising its accuracy. Through our experiments, we have demonstrated that E-CATBraTS, using the UCSF-PDGM and UPENN-GBM datasets, raises the segmentation accuracy and statistical stability, which helps in the early diagnosis of brain tumours, compared to the state-of-the-art models Swin UNetR, SegResNet, UNetR, and 3D CATBraTS. Furthermore, using the EGD dataset, our results were comparable to the highest accuracy obtained by Swin UNetR. A natural progression of this work is to assess the model in a real clinical setup, which could provide more reliable evidence. Further work will aim to evaluate E-CATBraTS by using other imaging acquisition methods, including computed tomography (CT) and positron emission tomography (PET). This approach will not only enhance the robustness of the findings but also ensure that the model can be adapted to various imaging technologies commonly used in clinical practice. Another aspect of future research includes investigating the accuracy and broader generalisability of E-CATBraTS for other biomedical segmentation tasks such as segmentation of lung, pancreatic, prostate, and breast tumours.

## Figures and Tables

**Table 1:** Quantitative evaluation of the proposed approach compared to the state-of-the-art models on UCSF-PDGM. Results presented as dice similarity coefficient (DSC) mean ± standard deviation (std). GRP is the number of subgroups in channel shuffle (highest accuracies are highlighted in bold).

		Network					
Metric	Region	SegResNet	UNETR	Swin UNetR	3D CATBraTS	E-CATBraTS (GRP = 2)	E-CATBraTS (GRP = 4)
DSC	Mean	0.673 ± 0.031	0.757 ± 0.045	0.749 ± 0.039	0.735 ± 0.038	0.761 ± 0.035	**0.795** ± 0.034
	TC	0.651 ± 0.073	0.694 ± 0.067	0.667 ± 0.048	0.667 ± 0.064	0.680 ± 0.044	**0.722** ± 0.0589
	WT	0.779 ± 0.027	0.833 ± 0.026	0.834 ± 0.033	0.802 ± 0.018	0.851 ± 0.023	**0.884** ± 0.013
	ET	0.588 ± 0.040	0.744 ± 0.054	0.748 ± 0.048	0.737 ± 0.045	0.753 ± 0.05	**0.778** ± 0.043
Jaccard	Mean	0.551 ± 0.028	0.653 ± 0.050	0.645 ± 0.041	0.624 ± 0.038	0.659 ± 0.036	**0.697** ± 0.039
	TC	0.54 ± 0.074	0.586 ± 0.073	0.55 ± 0.043	0.552 ± 0.064	0.568 ± 0.037	**0.612** ± 0.065
	WT	0.659 ± 0.031	0.735 ± 0.032	0.746 ± 0.04	0.69 ± 0.022	0.761 ± 0.0305	**0.803** ± 0.018
	ET	0.455 ± 0.037	0.639 ± 0.056	0.641 ± 0.05	0.628 ± 0.044	0.649 ± 0.052	**0.675** ± 0.046
Hausdorff	Mean	25.685 ± 4.238	25.755 ± 2.600	21.968 ± 5.591	25.783 ± 5.542	18.323 ± 5.883	**13.918** ± 2.482
	TC	22.404 ± 7.582	23.152 ± 4.408	23.909 ± 4.513	24.951 ± 5.816	20.768 ± 5.929	**16.553** ± 2.684
	WT	36.426 ± 5.929	33.393 ± 5.179	20.8 ± 7.744	30.48 ± 6.31	18.333 ± 6.156	**15.748** ± 4.457
	ET	18.226 ± 5.422	20.719 ± 5.665	21.194 ± 5.384	21.918 ± 6.532	15.866 ± 5.994	**9.453** ± 2.238

**Table 2:** Quantitative evaluation of proposed approach compared to the state-of-the-art models on UPENN-GBM. Results presented as dice similarity coefficient (DSC) mean ± standard deviation (std). GRP is the number of subgroups in channel shuffle (highest accuracies are highlighted in bold).

		Network					
**Metric**	**Region**	**SegResNet**	**UNETR**	**Swin UNetR**	**3D CATBraTS**	**E-CATBraTS** **(GRP = 2)**	**E-CATBraTS** **(GRP = 4)**
DSC	Mean	0.751 ± 0.009	0.856 ± 0.017	0.857 ± 0.019	0.824 ± 0.014	**0.871** ± 0.014	0.850 ± 0.009
	TC	0.789 ± 0.015	0.823 ± 0.024	0.811 ± 0.027	0.782 ± 0.021	**0.857** ± 0.02	0.802 ± 0.015
	WT	0.826 ± 0.008	0.891 ± 0.012	0.905 ± 0.013	0.854 ± 0.009	0.9 ± 0.012	**0.909** ± 0.009
	ET	0.637 ± 0.02	0.854 ± 0.02	0.855 ± 0.02	0.837 ± 0.019	**0.856** ± 0.015	0.839 ± 0.012
Jaccard	Mean	0.623 ± 0.01	0.765 ± 0.025	0.769 ± 0.028	0.719 ± 0.019	**0.790** ± 0.021	0.759 ± 0.012
	TC	0.672 ± 0.02	0.718 ± 0.034	0.705 ± 0.041	0.665 ± 0.028	**0.774** ± 0.028	0.693 ± 0.021
	WT	0.713 ± 0.01	0.813 ± 0.017	0.835 ± 0.019	0.753 ± 0.012	0.828 ± 0.016	**0.84** ± 0.014
	ET	0.485 ± 0.019	0.763 ± 0.03	**0.767** ± 0.03	0.739 ± 0.026	0.766 ± 0.021	0.744 ± 0.015
Hausdorff	Mean	13.649 ± 1.452	11.128 ± 4.426	7.320 ± 1.726	15.407 ± 7.867	**7.093** ± 5.05	7.905 ± 2.143
	TC	10.367 ± 1.794	11.872 ± 3.647	9.496 ± 1.36	13.768 ± 7.784	**7.776** ± 6.171	10.727 ± 1.614
	WT	19.904 ± 5.558	17.012 ± 11.923	**7.366** ± 6.142	24.189 ± 11.635	9.273 ± 5.196	7.406 ± 4.835
	ET	10.677 ± 1.793	4.501 ± 0.795	5.097 ± 1.154	8.263 ± 5.56	**4.23** ± 5.711	5.583 ± 0.773

**Table 3:** Dice similarity coefficient (DSC) mean ± standard deviation (std) on EGD (highest accuracies are highlighted in bold).

		Network			
**Metric**	**Region**	**SegResNet**	**Swin UNetR**	**3D CATBraTS**	**E-CATBraTS**
DSC	WT	0.738 ± 0.022	**0.774** ± 0.026	0.732 ± 0.032	0.768 ± 0.024
Jaccard	WT	0.625 ± 0.024	**0.661** ± 0.031	0.616 ± 0.034	0.658 ± 0.032
Hausdorff	WT	41.790 ± 8.125	38.313 ± 8.024	44.999 ± 7.836	**34.255**± 8.341

**Table 4:** Segmentation results of E-CATBraTS compared to the current state-of-the-art models trained on the BraTS 2021 and evaluated on the UCSF-PDGM datasets (highest accuracies are highlighted in bold).

		Network			
**Metric**	**Region**	**SegResNet**	**Swin UNetR**	**3D CATBraTS**	**E-CATBraTS**
DSC	Mean	0.724 ± 0.059	0.751 ± 0.047	**0.809** ± 0.033	0.770 ± 0.080
	TC	0.737 ± 0.084	0.682 ± 0.084	**0.784** ± 0.045	0.726 ± 0.102
	WT	0.818 ± 0.016	0.823 ± 0.021	**0.851** ± 0.03	0.802 ± 0.032
	ET	0.616 ± 0.087	0.748 ± 0.055	**0.792** ± 0.03	0.781 ± 0.154
Jaccard	Mean	0.606 ± 0.054	0.648 ± 0.053	**0.716** ± 0.04	0.658 ± 0.072
	TC	0.630 ± 0.077	0.576 ± 0.093	**0.690** ± 0.054	0.613 ± 0.098
	WT	0.709 ± 0.021	0.726 ± 0.025	**0.766** ± 0.034	0.696 ± 0.043
	ET	0.480 ± 0.073	0.643 ± 0.059	**0.692** ± 0.037	0.666 ± 0.132
Hausdorff	Mean	18.372 ± 9.995	31.620 ± 4.594	**10.034** ± 2.136	13.627 ± 2.284
	TC	12.671 ± 11.545	23.667 ± 6.574	**9.474** ± 2.286	9.723 ± 1.651
	WT	29.155 ± 7.718	50.733 ± 5.304	**13.709** ± 4.411	24.559 ± 6.921
	ET	13.291 ± 11.915	20.461 ± 7.383	6.919 ± 2.164	**6.598** ± 1.783

**Table 5:** Ablation study of the proposed model on four datasets: UCSF, UPENN, EGD, and BraTS 2021 (highest accuracies are highlighted in bold).

		Network			
Metric	Datasets	E-CATBraTS	No Attention	No Shuffle	No Shuffle No Attention
DSC	UCSF	**0.795** ± 0.034	0.745 ± 0.033	0.762 ± 0.031	0.719 ± 0.041
	UPENN	**0.871** ± 0.014	0.854 ± 0.010	0.859 ± 0.021	0.836 ± 0.02
	EGD	**0.768** ± 0.024	0.753 ± 0.037	0.755 ± 0.028	0.740 ± 0.034
	BraTS 2021	**0.770** ± 0.080	0.724 ± 0.047	0.707 ± 0.037	0.709 ± 0.052
Jaccard	UCSF	**0.697** ± 0.039	0.640 ± 0.031	0.657 ± 0.032	0.605 ± 0.044
	UPENN	**0.790** ± 0.021	0.765 ± 0.013	0.775 ± 0.030	0.741 ± 0.03
	EGD	**0.658** ± 0.032	0.639 ± 0.034	0.639 ± 0.032	0.625 ± 0.035
	BraTS 2021	**0.658** ± 0.072	0.615 ± 0.051	0.596 ± 0.035	0.603 ± 0.053
Hausdorff	UCSF	**13.918** ± 2.482	17.461 ± 2.600	25.196 ± 3.243	33.820 ± 4.635
	UPENN	7.093 ± 5.05	8.769 ± 2.250	**6.609** ± 1.898	6.711 ± 1.241
	EGD	**34.255** ± 8.341	40.145 ± 8.628	39.773 ± 7.702	46.015 ± 8.462
	BraTS 2021	**13.627** ± 2.284	23.249 ± 3.504	18.341 ± 2.524	14.272 ± 2.964

## Data Availability

These datasets were derived from the following resources available in the public domain: https://www.cancerimagingarchive.net/collection/ucsf-pdgm/, (accessed on 31 July 2024), https://www.cancerimagingarchive.net/collection/upenn-gbm/, (accessed on 31 July 2024) and http://braintumorsegmentation.org/, (accessed on 31 July 2024).

## References

[1] Kortz M.W., Lillehei K.O. (2023). Insular Cortex.

[2] Huisman T.A. (2009). Tumor-like lesions of the brain. Cancer Imaging.

[3] McFaline-Figueroa J.R., Lee E.Q. (2018). Brain Tumors. Am. J. Med..

[4] National Cancer Institute Adult Central Nervous System Tumors Treatment (PDQ®)–Patient Version—NCI, 2022. https://www.cancer.gov/types/brain/patient/adult-brain-treatment-pdq.

[5] Louis D.N., Perry A., Wesseling P., Brat D.J., Cree I.A., Figarella-Branger D., Hawkins C., Ng H.K., Pfister S.M., Reifenberger G. (2021). The 2021 WHO Classification of Tumors of the Central Nervous System: A summary. Neuro-Oncology.

[6] NHS England (2022). Survival by Cancer Group. https://digital.nhs.uk/data-and-information/publications/statistical/cancer-survival-in-england/cancers-diagnosed-2015-to-2019-followed-up-to-2020/survival-by-cancer-group.

[7] Hanna T.P., King W.D., Thibodeau S., Jalink M., Paulin G.A., Harvey-Jones E., O’Sullivan D.E., Booth C.M., Sullivan R., Aggarwal A. (2020). Mortality due to cancer treatment delay: Systematic review and meta-analysis. BMJ.

[8] McDonald R.J., Schwartz K.M., Eckel L.J., Diehn F.E., Hunt C.H., Bartholmai B.J., Erickson B.J., Kallmes D.F. (2015). The Effects of Changes in Utilization and Technological Advancements of Cross-Sectional Imaging on Radiologist Workload. Acad. Radiol..

[9] Villarini B., Asaturyan H., Kurugol S., Afacan O., Bell J.D., Thomas E.L. 3D Deep Learning for Anatomical Structure Segmentation in Multiple Imaging Modalities. Proceedings of the 2021 IEEE 34th International Symposium on Computer-Based Medical Systems (CBMS).

[10] Yamashita R., Nishio M., Do R.K.G., Togashi K. (2018). Convolutional neural networks: An overview and application in radiology. Insights Imaging.

[11] Albawi S., Mohammed T.A., Al-Zawi S. Understanding of a convolutional neural network. Proceedings of the 2017 International Conference on Engineering and Technology (ICET).

[12] Li Q., Cai W., Wang X., Zhou Y., Feng D.D., Chen M. Medical image classification with convolutional neural network. Proceedings of the 2014 13th International Conference on Control Automation Robotics & Vision (ICARCV).

[13] Tabrizchi H., Parvizpour S., Razmara J. (2023). An Improved VGG Model for Skin Cancer Detection. Neural Process. Lett..

[14] Cen L.P., Ji J., Lin J.W., Ju S.T., Lin H.J., Li T.P., Wang Y., Yang J.F., Liu Y.F., Tan S. (2021). Automatic detection of 39 fundus diseases and conditions in retinal photographs using deep neural networks. Nat. Commun..

[15] Vaswani A., Shazeer N., Parmar N., Uszkoreit J., Jones L., Gomez A.N., Kaiser L., Polosukhin I. (2017). Attention Is All You Need. arXiv.

[16] Dosovitskiy A., Beyer L., Kolesnikov A., Weissenborn D., Zhai X., Unterthiner T., Dehghani M., Minderer M., Heigold G., Gelly S. (2021). An Image is Worth 16x16 Words: Transformers for Image Recognition at Scale. arXiv.

[17] van Timmeren J.E., Cester D., Tanadini-Lang S., Alkadhi H., Baessler B. (2020). Radiomics in medical imaging—“How-to” guide and critical reflection. Insights Imaging.

[18] Ronneberger O., Fischer P., Brox T. (2015). U-Net: Convolutional Networks for Biomedical Image Segmentation. arXiv.

[19] Long J., Shelhamer E., Darrell T. (2015). Fully Convolutional Networks for Semantic Segmentation. arXiv.

[20] Myronenko A. (2018). 3D MRI Brain Tumor Segmentation Using Autoencoder Regularization. arXiv.

[21] He K., Zhang X., Ren S., Sun J. (2015). Deep Residual Learning for Image Recognition. arXiv.

[22] Bakas S., Reyes M., Jakab A., Bauer S., Rempfler M., Crimi A., Shinohara R.T., Berger C., Ha S.M., Rozycki M. (2019). Identifying the Best Machine Learning Algorithms for Brain Tumor Segmentation, Progression Assessment, and Overall Survival Prediction in the BRATS Challenge. arXiv.

[23] Ying X. (2019). An Overview of Overfitting and Its Solutions. J. Phys. Conf. Ser..

[24] Hatamizadeh A., Nath V., Tang Y., Yang D., Roth H., Xu D. (2022). Swin UNETR: Swin Transformers for Semantic Segmentation of Brain Tumors in MRI Images. arXiv.

[25] Liu Z., Lin Y., Cao Y., Hu H., Wei Y., Zhang Z., Lin S., Guo B. (2021). Swin Transformer: Hierarchical Vision Transformer Using Shifted Windows. arXiv.

[26] Isensee F., Petersen J., Klein A., Zimmerer D., Jaeger P.F., Kohl S., Wasserthal J., Koehler G., Norajitra T., Wirkert S. (2018). nnU-Net: Self-adapting Framework for U-Net-Based Medical Image Segmentation. arXiv.

[27] Isensee F., Jäger P.F., Full P.M., Vollmuth P., Maier-Hein K.H. (2020). nnU-Net for Brain Tumor Segmentation. arXiv.

[28] Wang W., Chen C., Ding M., Li J., Yu H., Zha S. (2021). TransBTS: Multimodal Brain Tumor Segmentation Using Transformer. arXiv.

[29] Goyal B., Dogra A., Agrawal S., Sohi B. (2018). Noise Issues Prevailing in Various Types of Medical Images. Biomed. Pharmacol. J..

[30] El Badaoui R., Coll E.B., Psarrou A., Villarini B. 3D CATBraTS: Channel Attention Transformer for Brain Tumour Semantic Segmentation. Proceedings of the 2023 IEEE 36th International Symposium on Computer-Based Medical Systems (CBMS).

[31] Lin M., Chen Q., Yan S. (2014). Network in Network. arXiv.

[32] Song Z., Qiu D., Zhao X., Lin D., Hui Y. (2023). Channel attention generative adversarial network for super-resolution of glioma magnetic resonance image. Comput. Methods Programs Biomed..

[33] Calabrese E., Villanueva-Meyer J.E., Rudie J.D., Rauschecker A.M., Baid U., Bakas S., Cha S., Mongan J.T., Hess C.P. (2022). The University of California San Francisco Preoperative Diffuse Glioma MRI Dataset. Radiol. Artif. Intell..

[34] Calabrese E., Villanueva-Meyer J., Rudie J., Rauschecker A., Baid U., Bakas S., Cha S., Mongan J., Hess C. (2023). The University of California San Francisco Preoperative Diffuse Glioma MRI (UCSF-PDGM). arXiv.

[35] Clark K., Vendt B., Smith K., Freymann J., Kirby J., Koppel P., Moore S., Phillips S., Maffitt D., Pringle M. (2013). The Cancer Imaging Archive (TCIA): Maintaining and Operating a Public Information Repository. J. Digit. Imaging.

[36] Bakas S., Sako C., Akbari H., Bilello M., Sotiras A., Shukla G., Rudie J.D., Flores Santamaria N., Fathi Kazerooni A., Pati S. (2021). Multi-Parametric Magnetic Resonance Imaging (mpMRI) Scans for De Novo Glioblastoma (GBM) Patients from the University of Pennsylvania Health System (UPENN-GBM). https://www.cancerimagingarchive.net/collection/upenn-gbm/.

[37] Bakas S., Sako C., Akbari H., Bilello M., Sotiras A., Shukla G., Rudie J.D., Santamaría N.F., Kazerooni A.F., Pati S. (2022). The University of Pennsylvania glioblastoma (UPenn-GBM) cohort: Advanced MRI, clinical, genomics, & radiomics. Sci. Data.

[38] van der Voort S.R., Incekara F., Wijnenga M.M.J., Kapsas G., Gahrmann R., Schouten J.W., Dubbink H.J., Vincent A.J.P.E., van den Bent M.J., French P.J. (2021). The Erasmus Glioma Database (EGD): Structural MRI scans, WHO 2016 subtypes, and segmentations of 774 patients with glioma. Data Brief.

[39] Marcus D.S., Olsen T.R., Ramaratnam M., Buckner R.L. (2007). The extensible neuroimaging archive toolkit. Neuroinformatics.

[40] Menze B.H., Jakab A., Bauer S., Kalpathy-Cramer J., Farahani K., Kirby J., Burren Y., Porz N., Slotboom J., Wiest R. (2014). The Multimodal Brain Tumor Image Segmentation Benchmark (BRATS). IEEE Trans. Med. Imaging.

[41] Bakas S., Akbari H., Sotiras A., Bilello M., Rozycki M., Kirby J.S., Freymann J.B., Farahani K., Davatzikos C. (2017). Advancing The Cancer Genome Atlas glioma MRI collections with expert segmentation labels and radiomic features. Sci. Data.

[42] Baid U., Ghodasara S., Mohan S., Bilello M., Calabrese E., Colak E., Farahani K., Kalpathy-Cramer J., Kitamura F.C., Pati S. (2021). The RSNA-ASNR-MICCAI BraTS 2021 Benchmark on Brain Tumor Segmentation and Radiogenomic Classification. arXiv.

[43] Cardoso M.J., Li W., Brown R., Ma N., Kerfoot E., Wang Y., Murrey B., Myronenko A., Zhao C., Yang D. (2022). MONAI: An Open-Source Framework for Deep Learning in Healthcare. arXiv.

[44] Loshchilov I., Hutter F. (2017). SGDR: Stochastic Gradient Descent with Warm Restarts. arXiv.

[45] Loshchilov I., Hutter F. (2019). Decoupled Weight Decay Regularization. arXiv.

[46] Milletari F., Navab N., Ahmadi S.A. V-Net: Fully Convolutional Neural Networks for Volumetric Medical Image Segmentation. Proceedings of the 2016 Fourth International Conference on 3D Vision (3DV).

[47] Zhang X., Zhou X., Lin M., Sun J. (2017). ShuffleNet: An Extremely Efficient Convolutional Neural Network for Mobile Devices. arXiv.

